# Caesium propano­ate monohydrate

**DOI:** 10.1107/S2056989020009639

**Published:** 2020-07-17

**Authors:** Erika Samolová, Jan Fábry

**Affiliations:** a Inst. of Physics of the Czech Academy of Sciences, Na Slovance 2, 182 21 Praha 8, Czech Republic

**Keywords:** crystal structure, hydrogen bonding, metal–organic compounds, Cambridge Structural Database, positional disorder, occupational disorder, hydrates

## Abstract

Caesium propano­ate monohydrate, Cs^+^·C_3_H_5_O_2_^−^·H_2_O, is composed of two symmetry-independent Cs^+^ cations, which are situated on the special position 4*e* of space group *P* 2_1_ *m*, one symmetry-independent propano­ate mol­ecule in a general position and a pair of water mol­ecules also situated on special position 4*e*. Two pairs of these symmetry-independent cations, four propano­ate mol­ecules and two pairs of symmetry-independent water mol­ecules form a repeat unit. These units form columns that are directed along the *c* axis and possess symmetry *mm*2. There are four such columns passing through each unit cell. Each column is inter­connected to its neighbours by four bifurcated three-centred O_w_—H⋯O_p_ (w = water, p = propano­ate) hydrogen bonds of moderate strength. There are also four intra­molecular O_w_—H⋯O_p_ hydrogen bonds of moderate strength within each column. One Cs^+^ cation is coordinated by six oxygen atoms (two water and four carboxyl­ate) in a trigonal–prismatic geometry, while the other Cs^+^ cation is coordinated by four water and four carboxyl­ate O atoms in a tetra­gonal–prismatic arrangement.

## Abstract 2

Caesium propano­ate monohydrate is composed of two symmetry-independent Cs^+^ cations, which are situated on special position 4*e*, one symmetry-independent propano­ate mol­ecule in a general position and a pair of water mol­ecules also situated on special position 4*e*. Two pairs of these symmetry-independent cations, four propano­ate mol­ecules and two pairs of symmetry-independent water mol­ecules form a repeat unit, which gives rise to columns running along the *c-*axis direction. These columns are held together by inter­molecular O_w_—H⋯O_p_ (w = water, p = propano­ate) hydrogen bonds of moderate strength, and other pairs of moderate intra­molecular hydrogen bonds reinforce the cohesion within the columnar unit.

## Chemical context   

No structure of a simple hydrated alkali propano­ate has been determined until now. (‘Simple’ means a structure where the constituting cation belongs just to one chemical species.) This is in contrast to alkali formates and acetates where water-free alkali salts and complexes with parent acids as well as hydrates are known. These structures show different structural motifs: Some of them are layered, such as lithium acetate dihydrate, LiC_2_H_3_O_2_·2H_2_O [refcode LIACET06 (Kearley *et al.*, 1996 ▸) in the Cambridge Structural Database, version 5.41, update of November 2019 (Groom *et al.*, 2016 ▸)], some are columnar including sodium di­hydrogen tri­acetate, NaC_2_H_3_O_2_·2C_2_H_4_O_2_ (NADHAC; Perotti & Tazzoli, 1981 ▸) while the cations and anions surround each other in the structure of *catena*-[bis­(μ_4_-acetato)­tetra­kis­(μ_3_-acetato)­bis­(μ_2_-acetato)­octa­aqua­octa­lith­ium] (UVELAJ; Martínez Casado *et al.*, 2011 ▸).
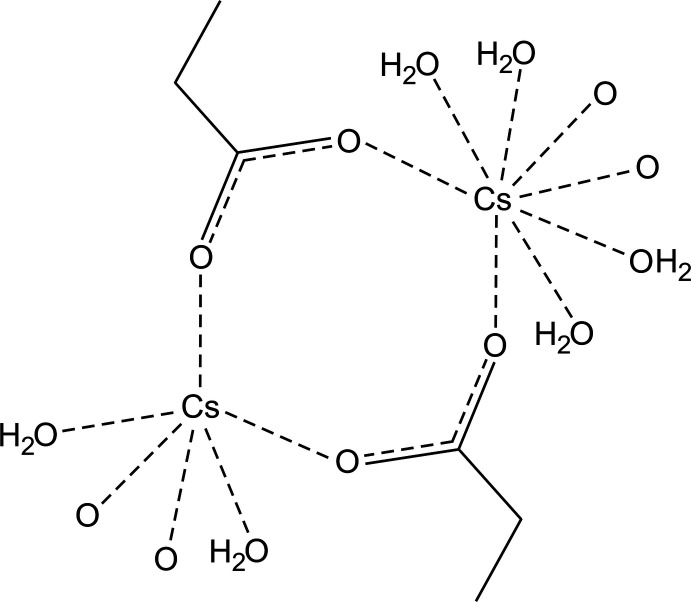


In the series of carb­oxy­lic acids with an increasing number of carbon atoms, it is propionic acid where the hydro­phobic properties start to be prominent. Formates and acetates are definitely distinct from propano­ates and other carboxyl­ates related to the acids C_*n*_H_2*n*+1_CO_2_ where *n* > 2. This is due to the longer and more voluminous organic chains in the latter compounds, which need space [*cf.* Duruz & Ubbelohde (1972 ▸) and Dumbleton & Lomer, 1965 ▸]. Cohesion is provided by van der Waals forces. Occasionally, positional disorder of these groups may take place. Dicalcium barium hexa­kis­(propano­ate) (Stadnicka & Glazer, 1980 ▸) can serve as an example.

The structural differences between alkali formates and acetates on one hand and simple alkali propano­ates such as Li(C_3_H_5_O_2_) (Martínez Casado *et al.*, 2009 ▸) and *M*(C_3_H_5_O_2_); *M* = Na, K, Rb, Cs (Fábry & Samolová, 2020 ▸) on the other reflect the chemical differences between these two groups of compounds. The latter structures are characterized by stacking of layers that are composed of a metal–oxygen bilayer surrounded by hydro­phobic layers comprising the ethyl groups. The cohesion forces between the hydro­phobic layers hold these structures together. The structure of the chemically related compound Tl(C_3_H_5_O_2_) (refcode WEWKAM; Martínez Casado *et al.*, 2010 ▸) is also a layered structure with three symmetry-independent cations.

Mirnaya *et al.* (1991 ▸) pointed out the tendency for various alkanoates to form hydrates. Such a case is reported in this study – see the *Synthesis and crystallization* section. It is of inter­est how strikingly different the title structure Cs(C_3_H_5_O_2_)·H_2_O is from Cs(C_3_H_5_O_2_) (Fábry & Samolová, 2020 ▸), despite the chemical similarity.

## Structural commentary   

**Figure 1:**
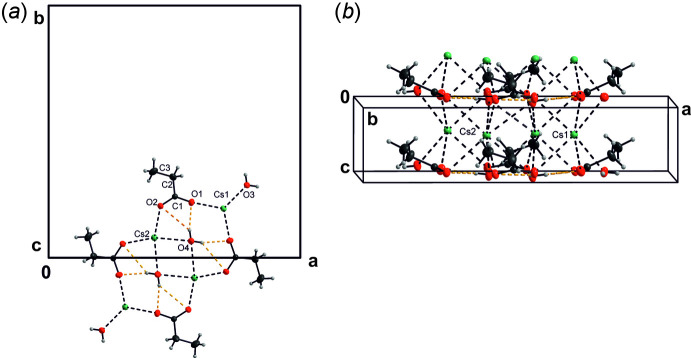
View (*DIAMOND*; Brandenburg, 2005 ▸) of the title motif along (*a*) the *c* axis and (*b*) the *b* axis. Displacement ellipsoids are shown at the 30% probability level: the cations and the O and C atoms are shown in green, red and grey, respectively. H atoms are shown as small light-grey spheres. The covalent bonds are represented by solid lines, Cs—O bonds by dashed black lines and hydrogen bonds by yellow dashed lines.

**Figure 2:**
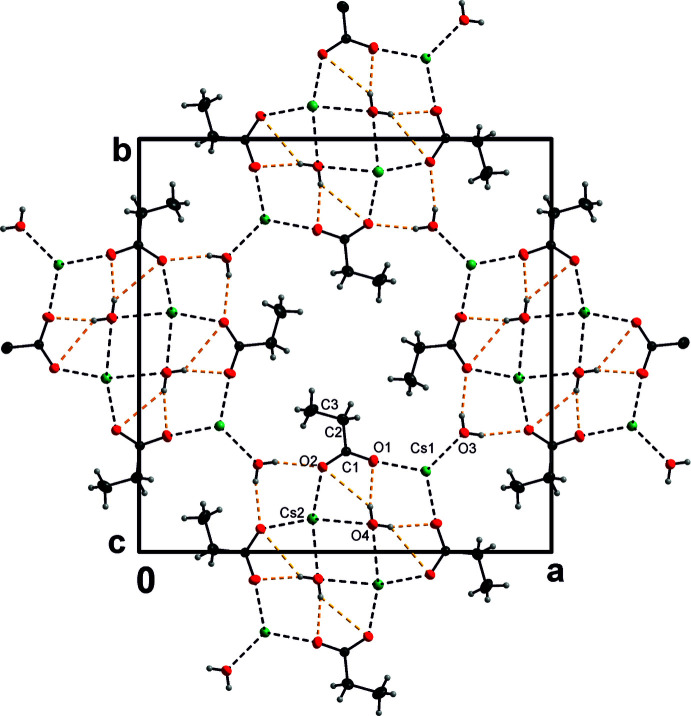
Packing of the title mol­ecules in the unit cell (*DIAMOND;* Brandenburg, 2005 ▸). Displacement ellipsoids are shown at the 30% probability level: the cations and the O and C atoms are shown in green, red and grey, respectively. H atoms are shown as small light-grey spheres. The covalent bonds are represented by solid lines, Cs—O bonds by dashed black lines and hydrogen bonds by yellow dashed lines.

The title structure confirms the tendency for various alkanoates to form hydrates, as noted by Mirnaya *et al.* (1991 ▸). Caesium propano­ate monohydrate is composed of two symmetry-independent Cs^+^ cations, which are situated on the special position 4*e*, *i.e.* on a symmetry plane, one symmetry-independent propano­ate mol­ecule in a general position and a pair of water mol­ecules also situated on special position 4*e* of space group *P* 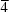
2_1_ *m*. Two pairs of these symmetry-independent cations, four propano­ate mol­ecules and two pairs of symmetry-independent water mol­ecules form a repeat unit. These units form columns along the *c-*axis direction (Fig. 1 ▸ *a*). The length of the repeat unit along the *c* axis corresponds to this unit-axis length. Each column has *mm*2 symmetry (Fig. 1 ▸ *b*). There are four such columns passing through each unit cell (Fig. 2 ▸). The columns are inter­connected by bifurcated three-centered O_w_—H⋯O_p_ (w = water, p = propano­ate) hydrogen bonds (Jeffrey, 1995 ▸), whose lengths and angles are quite different, but which are still of moderate strength (Gilli & Gilli, 2009 ▸); the donor is O4 and the donated hydrogen is H1*o*4 (Table 1 ▸). Each column thus donates four three-centered bifurcated hydrogen bonds (Jeffrey, 1995 ▸) to its neighbours (Fig. 2 ▸). There are also intra­molecular two-centered O_w_—H⋯O_p_ hydrogen bonds of moderate strength within each column in the structure; the donor is O3 and the donated atom is H1*o*3.

Cs1 is coordinated by six oxygen atoms (two of them are water O atoms and four are carboxyl­ate O atoms) in a trigonal–prismatic geometry, while Cs2 is in a less regular tetra­gonal–prismatic coordination environment (by four water and four carboxyl­ate oxygen atoms) (Fig. 2 ▸). The bond-valence sums (Brese & O’Keeffe, 1991 ▸) of Cs1 and Cs2 are 0.902 (3) and 0.997 (2) v.u., respectively. The fact that the cation with a smaller number of ligands that exhibits a regular coordination environment has a smaller bond-valence sum than that with the larger number of surrounding cations seems to be a peculiarity of the present structure, for example compared to β-K_2_SO_4_ compounds with two symmetry-independent cations (Fábry & Pérez-Mato, 1994 ▸). One has eleven ligands while the other has nine. The former has a more irregular coordination compared to the latter and its bond-valence sum is also lower than that of the latter cation. This example is a specific case that has been considered by Brown (1992 ▸): The larger coordination number usually results in the formation of a larger cavity around the cation. Stabilization of the cation causes the cation to shift towards some ligand. Such a shift contributes to irregularity of the coordination polyhedron with large numbers of ligands. Despite this stabilization, the bonding of a cation with a high coordination number tends to be lower than that of a cation with a low coordination number.

In contrast to the alkali propano­ates, *M*(C_*n*_H_2*n*+1_COO), the methyl­ene–methyl­ene, meth­yl–methyl carbon atoms are not in close contact in the title structure. The closest contact C2⋯C3^vi^, *i.e.* a methyl­ene–methyl contact is 3.961 (4) Å; symmetry code: (vi) −*y* + 1, *x*, −*z* + 1. This is related to the fact that no disorder of the ethyl groups is observed in the studied structure. At the same time, there are elongated voids in the *c*-axis direction that run parallel through the 4*d* positions and which are surrounded by the ethyl groups. The radius of the void is 1.381 Å while its height nearly corresponds to the *c* axis. The voids were calculated and depicted (see the supporting information) using *Mercury* 4.0 (Macrae *et al.*, 2020 ▸).

## Synthesis and crystallization   

The crystals formed spontaneously in a droplet from dissolved deliquescent crystals of Cs(C_3_H_5_O_2_) that otherwise have been grown from an aqueous solution of Cs_2_CO_3_ with a little excess of propionic acid (Fábry & Samolová, 2020 ▸).

## Structure determination and refinement   

Crystal data, data collection and structure refinement details are summarized in Table 2 ▸. The non-hydrogen atoms were determined by a charge-flipping method (Palatinus & Chapuis, 2007 ▸). The positions of the methyl­ene hydrogen atoms were calculated and refined under the following constraints: C—H = 0.97 Å with *U*_iso_(H) = 1.2*U*_eq_(C). The methyl hydrogen atoms were found in the difference electron-density maps and refined under the constraints C—H = 0.96 Å and *U*_iso_(H) = 1.5*U*_eq_(C). The water hydrogen atoms were also found in the difference electron density maps. The O—H distances were restrained to 0.820 (1) Å with *U*_iso_(H) = 1.5*U*_eq_(O). When the water H atoms were refined, the O—H_water_ distances converged to values of ∼0.78 Å. The structure was treated as an inversion twin. The Flack (1983 ▸) parameter is 0.03 (3).

## Supplementary Material

Crystal structure: contains datablock(s) global, I. DOI: 10.1107/S2056989020009639/dj2008sup1.cif

Structure factors: contains datablock(s) I. DOI: 10.1107/S2056989020009639/dj2008Isup2.hkl

Supporting information file. DOI: 10.1107/S2056989020009639/dj2008Isup3.smi

Supporting information file. DOI: 10.1107/S2056989020009639/dj2008Isup4.cml

View of the voids in the structure. DOI: 10.1107/S2056989020009639/dj2008sup5.docx

CCDC reference: 2016565

Additional supporting information:  crystallographic information; 3D view; checkCIF report

## Figures and Tables

**Table 1:** Hydrogen-bond geometry (Å, °)

*D*—H⋯*A*	*D*—H	H⋯*A*	*D*⋯*A*	*D*—H⋯*A*
O3—H1*o*3⋯O2^i^	0.820 (9)	1.988 (7)	2.783 (2)	163.3 (19)
O4—H1*o*4⋯O1^ii^	0.820 (7)	1.941 (6)	2.748 (2)	168.0 (5)
O4—H1*o*4⋯O2^ii^	0.820 (7)	2.646 (5)	3.293 (2)	137.0 (5)
O3—H1*o*3^iii^⋯O2^iv^	0.820 (9)	1.988 (7)	2.783 (2)	163.3 (19)
O4—H1*o*4^iii^⋯O1^v^	0.820 (7)	1.941 (6)	2.748 (2)	168.0 (5)
O4—H1*o*4^iii^⋯O2^v^	0.820 (7)	2.646 (5)	3.293 (2)	137.0 (5)

Symmetry codes: (i) 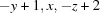; (ii) 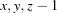; (iii) 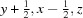; (iv) 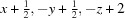; (v) 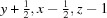.

**Table 2:** Experimental details

Crystal data	
Chemical formula	Cs^+^·C_3_H_5_O_2_^−^·H_2_O
*M*_r_	224
Crystal system, space group	Tetragonal, *P* 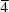 2_1_ *m*
Temperature (K)	230
*a*, *c* (Å)	17.7764 (3), 4.2223 (1)
*V* (Å^3^)	1334.25 (4)
*Z*	8
Radiation type	Mo *K*α
μ (mm^−1^)	5.47
Crystal size (mm)	0.46 × 0.04 × 0.03

Data collection	
Diffractometer	Bruker D8 VENTURE Kappa Duo PHOTON 100 CMOS
Absorption correction	Multi-scan (*SADABS*; Bruker, 2017 ▸)
*T*_min_, *T*_max_	0.190, 0.866
No. of measured, independent and observed [*I* > 3σ(*I*)] reflections	12844, 2019, 1872
*R*_int_	0.021
(sin θ/λ)_max_ (Å^−1^)	0.703

Refinement	
*R*[*F* > 3σ(*F*)], *wR*(*F*), *S*	0.014, 0.037, 1.10
No. of reflections	2019
No. of parameters	78
No. of restraints	4
H-atom treatment	H atoms treated by a mixture of independent and constrained refinement
Δρ_max_, Δρ_min_ (e Å^−3^)	0.24, −0.33
Absolute structure	Flack (1983 ▸), 831 Friedel pairs
Absolute structure parameter	0.03 (3)

Computer programs: *Instrument Service* and *SAINT* (Bruker, 2017 ▸), *SUPERFLIP* (Palatinus & Chapuis, 2007 ▸) and *JANA2006* (Petříček *et al.*, 2014 ▸), *DIAMOND* (Brandenburg, 2005 ▸); extinction correction according to Becker & Coppens (1974 ▸).

## supplementary crystallographic information

### Crystal data

**Table:**

Cs^+^·C_3_H_5_O_2_^−^·H_2_O	There have been used diffractions with I/σ(I)>20 for the unit cell determination.
*M**_r_* = 224	*D*_x_ = 2.230 Mg m^−^^3^
Tetragonal, *P*42_1_*m*	Mo *K*α radiation, λ = 0.71073 Å
Hall symbol: P -4 2ab	Cell parameters from 9815 reflections
*a* = 17.7764 (3) Å	θ = 2.3–30.0°
*c* = 4.2223 (1) Å	µ = 5.47 mm^−^^1^
*V* = 1334.25 (4) Å^3^	*T* = 230 K
*Z* = 8	Needle, colourless
*F*(000) = 832	0.46 × 0.04 × 0.03 mm

### Data collection

**Table:**

Bruker D8 VENTURE Kappa Duo PHOTON 100 CMOS diffractometer	2019 independent reflections
Radiation source: IµS micro-focus sealed tube	1872 reflections with *I* > 3σ(*I*)
Quazar Mo multilayer optic monochromator	*R*_int_ = 0.021
φ and ω scans	θ_max_ = 30.0°, θ_min_ = 2.3°
Absorption correction: multi-scan (*SADABS*; Bruker, 2017)	*h* = −24→24
*T*_min_ = 0.190, *T*_max_ = 0.866	*k* = −19→24
12844 measured reflections	*l* = −5→4

### Refinement

**Table:**

Refinement on *F*^2^	H atoms treated by a mixture of independent and constrained refinement
*R*[*F* > 3σ(*F*)] = 0.014	Weighting scheme based on measured s.u.'s *w* = 1/(σ^2^(*I*) + 0.0004*I*^2^)
*wR*(*F*) = 0.037	(Δ/σ)_max_ = 0.042
*S* = 1.10	Δρ_max_ = 0.24 e Å^−^^3^
2019 reflections	Δρ_min_ = −0.33 e Å^−^^3^
78 parameters	Extinction correction: B-C type 1 Lorentzian isotropic (Becker & Coppens, 1974)
4 restraints	Extinction coefficient: 330 (110)
22 constraints	Absolute structure: Flack (1983), 831 Friedel pairs
Primary atom site location: charge flipping	Absolute structure parameter: 0.03 (3)

### Fractional atomic coordinates and isotropic or equivalent isotropic displacement parameters (Å^2^)

**Table:**

	*x*	*y*	*z*	*U*_iso_*/*U*_eq_	
Cs1	0.695062 (6)	0.195062 (6)	0.48412 (6)	0.03299 (4)	
Cs2	0.420270 (6)	0.079730 (6)	0.51015 (5)	0.03461 (4)	
O1	0.56778 (8)	0.21997 (9)	0.9877 (8)	0.0493 (5)	
O2	0.44454 (7)	0.20549 (7)	1.0090 (6)	0.0395 (4)	
O3	0.78820 (8)	0.28820 (8)	0.9682 (10)	0.0442 (7)	
O4	0.56624 (9)	0.06624 (9)	0.0554 (10)	0.0534 (9)	
C1	0.50192 (12)	0.24285 (12)	0.9444 (5)	0.0310 (6)	
C2	0.49413 (16)	0.32018 (16)	0.7976 (7)	0.0549 (9)	
C3	0.41676 (19)	0.34209 (18)	0.6915 (8)	0.0631 (11)	
H1c2	0.513302	0.35775	0.943391	0.0658*	
H2c2	0.528887	0.324875	0.621629	0.0658*	
H1c3	0.383944	0.344695	0.871774	0.0947*	
H2c3	0.418684	0.390343	0.589772	0.0947*	
H3c3	0.398058	0.305262	0.545034	0.0947*	
H1o3	0.7817 (4)	0.3335 (4)	0.994 (9)	0.0662*	
H1o4	0.5599 (3)	0.1117 (3)	0.035 (8)	0.0801*	

### Atomic displacement parameters (Å^2^)

**Table:**

	*U*^11^	*U*^22^	*U*^33^	*U*^12^	*U*^13^	*U*^23^
Cs1	0.03383 (7)	0.03383 (7)	0.03130 (9)	−0.00310 (6)	−0.00090 (7)	−0.00090 (7)
Cs2	0.03802 (7)	0.03802 (7)	0.02778 (8)	−0.00027 (6)	0.00236 (8)	−0.00236 (8)
O1	0.0321 (7)	0.0484 (8)	0.0675 (11)	0.0054 (6)	0.0002 (13)	0.0072 (13)
O2	0.0324 (6)	0.0340 (7)	0.0520 (9)	−0.0018 (5)	0.0050 (10)	0.0042 (11)
O3	0.0348 (6)	0.0348 (6)	0.0629 (18)	−0.0038 (8)	−0.0063 (12)	−0.0063 (12)
O4	0.0372 (7)	0.0372 (7)	0.086 (2)	−0.0007 (9)	0.0092 (11)	0.0092 (11)
C1	0.0314 (9)	0.0300 (9)	0.0316 (12)	0.0010 (8)	0.0005 (9)	0.0018 (9)
C2	0.0470 (15)	0.0427 (15)	0.0749 (18)	−0.0065 (12)	−0.0065 (14)	0.0230 (14)
C3	0.070 (2)	0.0518 (18)	0.067 (2)	0.0210 (16)	−0.0106 (17)	0.0148 (15)

### Geometric parameters (Å, º)

**Table:**

O1—C1	1.253 (3)	Cs1—O3	3.108 (3)
O2—C1	1.247 (3)	Cs2—O2	3.102 (2)
C1—C2	1.514 (4)	Cs2—O2^iv^	3.108 (2)
C2—C3	1.498 (4)	Cs2—O2^v^	3.102 (2)
C2—H1c2	0.97	Cs2—O3^vi^	3.886 (3)
C2—H2c2	0.97	Cs2—O3^vii^	3.984 (3)
C3—H1c3	0.96	Cs2—O4	3.237 (3)
C3—H2c3	0.96	Cs2—O4^viii^	3.477 (3)
C3—H3c3	0.96	Cs2—O4^ix^	3.237 (3)
Cs1—O1^i^	3.116 (2)	Cs2—O4^x^	3.477 (3)
Cs1—O1	3.136 (2)	O3—H1o3	0.820 (9)
Cs1—O1^ii^	3.116 (2)	O3—H1o3^iii^	0.820 (9)
Cs1—O1^iii^	3.136 (2)	O4—H1o4	0.820 (7)
Cs1—O3^i^	3.198 (3)	O4—H1o4^iii^	0.820 (7)
			
O1^i^—Cs1—O1	84.96 (7)	O2^v^—Cs2—O4^x^	59.75 (5)
O1^i^—Cs1—O1^ii^	75.75 (6)	O3^vi^—Cs2—O3^vii^	64.87 (8)
O1^i^—Cs1—O1^iii^	131.38 (5)	O3^vi^—Cs2—O4	102.75 (7)
O1^i^—Cs1—O3^i^	81.03 (6)	O3^vi^—Cs2—O4^viii^	146.78 (4)
O1^i^—Cs1—O3	138.89 (5)	O3^vi^—Cs2—O4^ix^	102.75 (7)
O1^i^—Cs1—O4	46.47 (5)	O3^vi^—Cs2—O4^x^	146.78 (4)
O1—Cs1—O1^ii^	131.38 (5)	O3^vii^—Cs2—O4	147.56 (4)
O1—Cs1—O1^iii^	75.17 (6)	O3^vii^—Cs2—O4^viii^	96.57 (7)
O1—Cs1—O3^i^	139.66 (4)	O3^vii^—Cs2—O4^ix^	147.56 (4)
O1—Cs1—O3	82.14 (7)	O3^vii^—Cs2—O4^x^	96.57 (7)
O1—Cs1—O4	88.43 (6)	O4—Cs2—O4^viii^	77.84 (8)
O1^ii^—Cs1—O1^iii^	84.96 (7)	O4—Cs2—O4^ix^	61.92 (5)
O1^ii^—Cs1—O3^i^	81.03 (6)	O4—Cs2—O4^x^	106.39 (6)
O1^ii^—Cs1—O3	138.89 (5)	O4^viii^—Cs2—O4^ix^	106.39 (6)
O1^ii^—Cs1—O4	46.47 (5)	O4^viii^—Cs2—O4^x^	57.23 (5)
O1^iii^—Cs1—O3^i^	139.66 (4)	O4^ix^—Cs2—O4^x^	77.84 (8)
O1^iii^—Cs1—O3	82.14 (7)	Cs1—O1—Cs1^viii^	84.96 (4)
O1^iii^—Cs1—O4	88.43 (6)	Cs2—O2—Cs2^viii^	85.68 (3)
O3^i^—Cs1—O3	84.05 (8)	Cs1—O3—Cs1^viii^	84.05 (4)
O3^i^—Cs1—O4	107.86 (8)	Cs1—O3—Cs2^xi^	107.57 (11)
O3—Cs1—O4	168.08 (8)	Cs1—O3—Cs2^xii^	172.44 (11)
O2^i^—Cs2—O2	85.68 (6)	Cs1^viii^—O3—Cs2^xi^	168.38 (11)
O2^i^—Cs2—O2^iv^	74.70 (5)	Cs1^viii^—O3—Cs2^xii^	103.51 (11)
O2^i^—Cs2—O2^v^	131.48 (4)	H1o3—O3—H1o3^iii^	105.0 (10)
O2^i^—Cs2—O3^vi^	45.21 (4)	Cs1—O4—Cs2^i^	137.82 (6)
O2^i^—Cs2—O3^vii^	91.97 (5)	Cs1—O4—Cs2	99.19 (9)
O2^i^—Cs2—O4	62.50 (5)	Cs1—O4—Cs2^xiii^	137.82 (6)
O2^i^—Cs2—O4^viii^	113.38 (5)	Cs1—O4—Cs2^ix^	99.19 (9)
O2^i^—Cs2—O4^ix^	99.35 (7)	Cs2^i^—O4—Cs2	77.84 (4)
O2^i^—Cs2—O4^x^	167.93 (4)	Cs2^i^—O4—Cs2^xiii^	70.40 (6)
O2—Cs2—O2^iv^	131.48 (4)	Cs2^i^—O4—Cs2^ix^	120.22 (5)
O2—Cs2—O2^v^	74.89 (5)	Cs2—O4—Cs2^xiii^	120.22 (5)
O2—Cs2—O3^vi^	90.09 (6)	Cs2—O4—Cs2^ix^	76.52 (7)
O2—Cs2—O3^vii^	44.10 (4)	Cs2^xiii^—O4—Cs2^ix^	77.84 (4)
O2—Cs2—O4	110.17 (5)	H1o4—O4—H1o4^iii^	105.0 (7)
O2—Cs2—O4^viii^	59.75 (5)	O1—C1—O2	124.0 (2)
O2—Cs2—O4^ix^	166.01 (5)	O1—C1—C2	116.1 (2)
O2—Cs2—O4^x^	94.51 (7)	O2—C1—C2	119.9 (2)
O2^iv^—Cs2—O2^v^	85.68 (6)	C1—C2—C3	116.3 (2)
O2^iv^—Cs2—O3^vi^	45.21 (4)	C1—C2—H1c2	109.47
O2^iv^—Cs2—O3^vii^	91.97 (5)	C1—C2—H2c2	109.47
O2^iv^—Cs2—O4	99.35 (7)	C3—C2—H1c2	109.47
O2^iv^—Cs2—O4^viii^	167.93 (4)	C3—C2—H2c2	109.47
O2^iv^—Cs2—O4^ix^	62.50 (5)	H1c2—C2—H2c2	101.72
O2^iv^—Cs2—O4^x^	113.38 (5)	C2—C3—H1c3	109.47
O2^v^—Cs2—O3^vi^	90.09 (6)	C2—C3—H2c3	109.47
O2^v^—Cs2—O3^vii^	44.10 (4)	C2—C3—H3c3	109.47
O2^v^—Cs2—O4	166.01 (5)	H1c3—C3—H2c3	109.47
O2^v^—Cs2—O4^viii^	94.51 (7)	H1c3—C3—H3c3	109.47
O2^v^—Cs2—O4^ix^	110.17 (5)	H2c3—C3—H3c3	109.47

Symmetry codes: (i) *x*, *y*, *z*−1; (ii) *y*+1/2, *x*−1/2, *z*−1; (iii) *y*+1/2, *x*−1/2, *z*; (iv) −*y*+1/2, −*x*+1/2, *z*−1; (v) −*y*+1/2, −*x*+1/2, *z*; (vi) *y*, −*x*+1, −*z*+1; (vii) *y*, −*x*+1, −*z*+2; (viii) *x*, *y*, *z*+1; (ix) −*x*+1, −*y*, *z*; (x) −*x*+1, −*y*, *z*+1; (xi) −*y*+1, *x*, −*z*+1; (xii) −*y*+1, *x*, −*z*+2; (xiii) −*x*+1, −*y*, *z*−1.

### Hydrogen-bond geometry (Å, º)

**Table:**

*D*—H···*A*	*D*—H	H···*A*	*D*···*A*	*D*—H···*A*
O3—H1*o*3···O2^xii^	0.820 (9)	1.988 (7)	2.783 (2)	163.3 (19)
O4—H1*o*4···O1^i^	0.820 (7)	1.941 (6)	2.748 (2)	168.0 (5)
O4—H1*o*4···O2^i^	0.820 (7)	2.646 (5)	3.293 (2)	137.0 (5)
O3—H1*o*3^iii^···O2^xiv^	0.820 (9)	1.988 (7)	2.783 (2)	163.3 (19)
O4—H1*o*4^iii^···O1^ii^	0.820 (7)	1.941 (6)	2.748 (2)	168.0 (5)
O4—H1*o*4^iii^···O2^ii^	0.820 (7)	2.646 (5)	3.293 (2)	137.0 (5)

Symmetry codes: (i) *x*, *y*, *z*−1; (ii) *y*+1/2, *x*−1/2, *z*−1; (iii) *y*+1/2, *x*−1/2, *z*; (xii) −*y*+1, *x*, −*z*+2; (xiv) *x*+1/2, −*y*+1/2, −*z*+2.
