# Supplementary material for: Caesium propano­ate monohydrate
Source: Acta Crystallogr E Crystallogr Commun. 2020 Jul 17;76(Pt 8):1307–10. doi: 10.1107/S2056989020009639 (PMC7405577; doi:10.1107/S2056989020009639)
Supplement: Supplementary file 5 [file e-76-01307-sup5.docx]

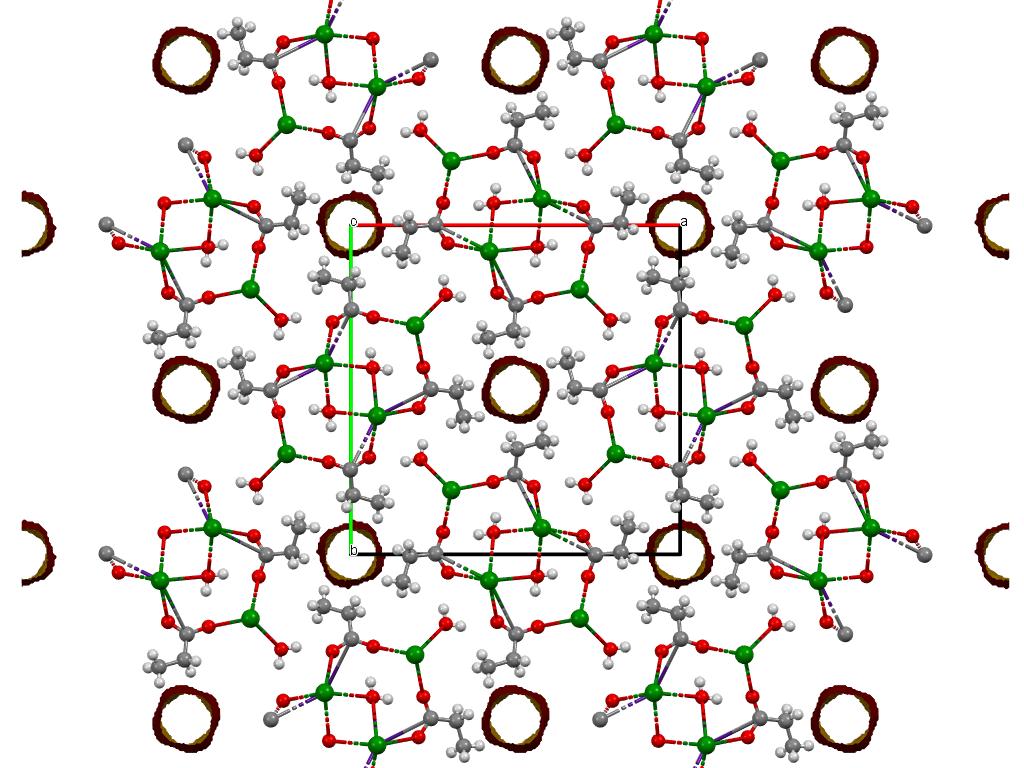


Voids (in brown) shown in the structure viewed along the axis *c* (Mercury, Macrae *et al*. 2020). The radius of the void shown here is 1.0 Å. Cs: green, O: red, C: gray, H: light gray.
